# Development of Functional Acid Curd Cheese (Tvarog) with Antioxidant Activity Containing Astaxanthin from Shrimp Shells Preliminary Experiment

**DOI:** 10.3390/foods10040895

**Published:** 2021-04-19

**Authors:** Izabela Dmytrów, Mariusz Szymczak, Katarzyna Szkolnicka, Patryk Kamiński

**Affiliations:** Department of Toxicology, Dairy Technology and Food Storage, Faculty of Food Science and Fisheries, West Pomeranian University of Technology in Szczecin, Papieża Pawła VI Street No. 3, 71-459 Szczecin, Poland; izabela.dmytrow@zut.edu.pl (I.D.); katarzyna.szkolnicka@zut.edu.pl (K.S.); patryk-kaminski@zut.edu.pl (P.K.)

**Keywords:** dairy product, astaxanthin, colour, acidity, lipids, DPPH, oxidation, storage

## Abstract

The food industry is looking for natural additives to improve acid curd cheese (tvarog), while shrimp by-products are being wasted. The concentrated astaxanthin lipid preparation (ALP) was recovered from shrimp shells and added (0%, 0.25%, 0.5% and 1%) to tvarogs stored up to 4 weeks at 5 ± 1 °C. The addition of ALP increased the lipid content and decreased the moisture in cheese. Water activity, acidity and hardness of tvarogs differed significantly between cheese variants. The cheeses with ALP had more stable and lower pH after 4 weeks of storage, and higher titratable acidity immediately after ALP addition. The 0–0.5% ALP samples had the same level and changes in lipid oxidation, while the 1% ALP cheese had more stable thiobarbituric acid values during storage. This may be due to several times greater antioxidant activity (DPPH assay) in the cheese with the highest ALP addition. The addition of astaxanthin had create popular salmon colour and improved objective colour parameters of the cheeses. The best sensory features had 0.5% ALP sample. A higher addition of astaxanthin preparation caused a foreign aftertaste. The use of astaxanthin from shrimp shells to acid curd cheeses enables the creation of new functional properties that are increasingly popular with consumers.

## 1. Introduction

Dairy products, including acid curd cheese (tvarog) in the form of wedges and slices, cottage cheese, and spreads for bread, account for a large amount of consumption worldwide. Tvarogs belong to the group of natural and unprocessed foods, for which no preservatives and artificial colours are used [1]. Seniors are more likely to choose traditional acid curd cheese without additives, while younger consumers are more likely to choose tvarog with new functional properties. Therefore, the dairy industry is looking for methods to improve the functional characteristics of this type of cheese by using, among others, vegetable additives and spices. These ingredients improve sensory characteristics, but mostly the functional value of cheese remains unchanged. Natural ingredients from food industry by-products can be a solution to the problem.

Shrimp shell waste has been a growing problem of environmental pollution and resource wastage for years [2]. Shrimp shells are important and the cheapest sources of natural astaxanthin and its esters as major pigments with great economic and social benefits [3]. Astaxanthin is a polar xanthophyll ketocarotenoid synthesized by plants and microorganisms but is mainly found in aquatic animals such as crustaceans, salmon, and trout [4,5]. Like other carotenoids, it is lipid-soluble but does not convert to vitamin A (retinol) in the human body.

Almost 90% of astaxanthin formulations available in the market contain a synthetic pigment that lacks bioactive properties [6]. Other formulations contain natural astaxanthin extracted from the unicellular algae *Hematococcus pluvialis* and various crustacean species such as the shrimp *Pandalus borealis*, as well as the yeast *Xanthophyllomyces dendrorhous* [7,8]. Natural astaxanthin has 10 times stronger antioxidant properties than other carotenoids [9] and is 500 times more effective than vitamin C and E [10]. It has beneficial effects that support human health, including mitigation of oxidative stress, inhibition of low-density lipoprotein (LDL) oxidation, enhancement of immune response, anti-inflammatory and anti-aging properties [11]. Astaxanthin also exhibits antimicrobial activity against spoilage and pathogenic bacteria found in food [12]. In the United States, it is approved as a food colorant (or colour additive) for special uses in animal and fish foods [13].

Many methods for astaxanthin extraction have been investigated so far, in which organic solvents have been most used [14,15]. Ethanol was found to be suitable for astaxanthin due to its safety, efficiency and easy separation [6,14,16]. The ethanolic extract of astaxanthin was used to enrich yogurt as an optional functional food, but in an encapsulated form, thus astaxanthin had no effect on food ingredients [17]. On the other hand, the use of astaxanthin directly in dairy products may be limited because this pigment is more soluble in long-chain lipids than in short-chain dairy lipids. Casein as a stabilizing colloid and lactose as a plastificator may protect and increase the stability of astaxanthin when exposed to oxygen and light [18,19]. Mezguita et al. [20] reported that astaxanthin oleoresin is well dispersed by the reducing of milk pH to 5.2, near the casein isoelectric point. Thus, the pH value of acid curd cheese (tvarog) may promote the biological activity of astaxanthin.

Therefore, the aim of this study was to investigate the effect of the addition of astaxanthin in the form of a lipid preparation obtained from shrimp shells on the nutritional value, sensory characteristics, functional properties and shelf life of cold-stored acid curd cheese.

## 2. Materials and Methods

### 2.1. Astaxanthin of Lipid Preparation (ALP) 

Frozen shells after hand peeling of the shrimp (*Pandalus borealis*) were obtained from the fish industry. The shells were dried at 40 °C for 24 h and grinded (Kenwood, CH250) to obtain a shell powder containing 3.8% moisture. Ethanol (99.8%) was added to the shell powder at a ratio of 1:10 (m:v), homogenized for 15 s at 28,000 rpm and centrifuged for 10 min at 9000× *g* at 4 °C. The supernatant was concentrated under vacuum at 50 °C until a constant volume of lipid fraction was obtained. The resulting astaxanthin lipid preparation (ALP) was stored for 2 days at −20 °C before cheese production.

### 2.2. Acid Curd Cheese (ACC) with Astaxanthin

Full-fat high-pasteurized (85 °C, 15 s) and homogenized (15 MPa, 55 °C) cow’s milk containing 3.2% fat, 3.1% protein and 4.4% lactose were purchased at a local market. The acid curd cheese (ACC) was produced by traditional technology [21,22], using a freeze-dried DVS starter to direct inoculation a milk. The starter culture contained *Lactococcus lactis subsp. cremoris, Lactococcus lactis subsp. lactis, Lactococcus lactis subsp. lactis biovar diacetylactis, Leuconostoc mesenteroides subsp. cremoris* (CHN-19, Flora Danica by Chr Hansen, Hoersholm, Denmark).

The production of the ACC in laboratory conditions started with heating the milk to 23 °C and adding 2.5 % (v:v) of the activated starter. The inoculated milk was incubated (23 °C, 12 h) until the curd reached pH 4.5. The curd was gently heated to separate from the walls of the cheese tub, then was cut into cuboids with dimensions of approximately 120 × 120 mm, gently mixed and gradually heated (1 °C/10 min) to 40 °C in the center in order to intensify the separation of whey. The curd mass was divided into disposable, polyethylene cheese cloths and allowed to drain. The ACCs obtained were pressed with a laboratory press for 45 min (1 kg per 1 kg of cheese).

Four variants of ACC samples were performed: one control sample and three experimental samples. The control sample was acid curd cheese without astaxanthin extract (0%). Astaxanthin lipid preparation (ALP) was added to the experimental tvarog at 0.25%, 0.5% and 1% (w:w). In this way, 3 variants of cheese with the addition of astaxanthin were obtained, i.e., 0.25% ALP, 0.5% ALP and 1% ALP. All samples were stirred for 2 min at 100 rpm. The individual ACC variants were divided into 50 smaller samples (100 g each), which were stored at 5 ± 1 °C in the dark in sterile and tightly sealed plastic containers. Analyses were performed immediately after the addition of astaxanthin (day 0) and at weekly intervals during a four-week storage period (28 days). Samples for testing were taken randomly and were kept for 10 min at room temperature before the analyses.

### 2.3. Moisture, Water Activity, PH and Titratable Acidity

Moisture content was measured using a drying method at 105 °C [23]. Water activity (a_w_) in the samples was measured with a HygroLab C1 hygrometer (Rotronic, Bassersdorf, Switzerland), using disposable cups. Measurements were carried out at a temperature of 23 °C. The pH was measured in the mixture obtained by the addition of 50 mL of distilled water to 50 g of sample and homogenizing the mixture at 4000 rpm for 20 s [24,25]. Measurements were made with a pH meter (AD 12, Adwa, Romania). The samples were analyzed for titratable acidity according to AOAC [24]. Titratable acidity was expressed as lactic acid percentage. 

### 2.4. Lipids, Astaxanthin Content and Thiobarbituric Acid Values

Lipids from ACC samples were extracted using the method of Bligh and Dyer [26]. The chloroform extract was used to determine (i) the lipids content after chloroform evaporation, (ii) astaxanthin content at 470 nm [9], (iii) the degree of lipid oxidation against thiobarbituric acid (TBA) and expressed in mg of malondialdehyde (MDA) per kg of tvarog [27].

### 2.5. Determination of DPPH-Free Radical-Scavenging Activity

The antioxidant activity of tvarogs with and without astaxanthin preparation was determined using DPPH (2,2-diphenyl-1-picryl-hydrazyl) at 570 nm. One milliliter of 0.25 mM DPPH solution in methanol was added to 1 mL of methanol extract of tvarog. The mixture was agitated using a vortex for 1 min and then left to stand at room temperature for 30 min in the darkness, and its absorbance was read at 517 nm. Then, the scavenging activity (%) of the mixture was evaluated as previously mentioned [28].

### 2.6. Colour Analysis

The colour of ACC was assessed with an objective method using colorimeter WR 18 (FRU^®^, Shenzhen Wave Optoelectronics Technology Co., Ltd., Shenzhen, China) [29], based on white standard tile (L* = +92.4; a* = −0.04; b* = +1.9) and CIE L* a* b*, illuminant D65, observer 10°, illumination mode d/8 and caliber 8 mm. The hue (h) and (C) chroma of colour were calculated according to [30,31]. The colour difference (ΔE = [(ΔL)^2^ + (Δa)^2^ + (Δb)^2^]^0.5^) was calculated for average value L* a* b*, where ΔL, Δa, and Δb are, respectively, the differences in the *L*, *a*, and *b,* values between the individual samples of the ACC.

### 2.7. Hardness Analysis

Hardness was determined with a TA-XT Plus Texture Analyzer (Stable Micro Systems, Godalming, UK). The samples were double-penetrated with a pressure of 1G and a velocity of 5 m/s up to a depth of 20 mm. The diameter of the aluminum probe was 6 mm [32].

### 2.8. Sensory Evaluation

Twelve participants (men and women) of various ages, examined for sensitivity of taste and experience in sensory evaluation of dairy products, took part in the sensory evaluation. Panelists were asked to indicate how much they liked or disliked each variant of ACC on a 5-point hedonic scale (5 = like extremely; 1 = dislike extremely). Flavour, odour, colour and consistency were evaluated according to standards ISO 22935-2:2013-07 [33] and ISO 22935-3:2013-07 [34]. The mean scores for each attribute to calculate overall sensory quality was used. The variance range in Figure 6 is for panel member assessment. The samples used for the analysis were selected randomly. The results for each descriptor were added together and were expressed as an arithmetic mean. The evaluation was carried out in a room that was free of any foreign odours; each panelist had a separate test stand and distilled water to rinse the mouth.

### 2.9. Statistical Analysis

The experiment was performed in one repetition, and all analyses were done in triplicate. Mean values and standard deviations were calculated [35]. The results were subjected to a statistical analysis carried out using Microsoft Excel software, using the Tukey test and a two-factor analysis of variance with repetition (ANOVA). All the tests were performed at a significance level of α = 0.05.

## 3. Results and Discussion

The amount of astaxanthin preparation addition was estimated on preliminary tests, in which changes in salmon colour, odour and taste of the ACC were evaluated. Doses lower than 0.25% were not noticeable to the naked eye, while an addition of more than 1% resulted in an excessively intense unnatural colour of the tvarog and a distinct foreign aftertaste.

**Figure 1:**
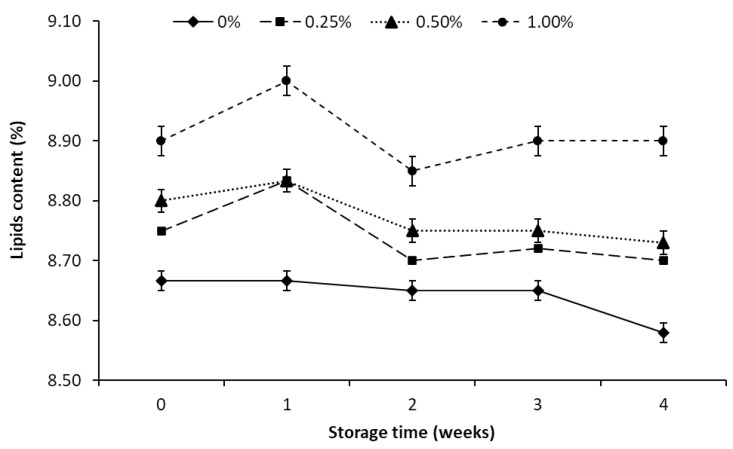
Lipids content in acid curd cheese with different addition (0–1%) of astaxanthin lipid preparation (ALP) during 4 weeks of refrigerated storage (5 ± 1 °C).

The control sample contained on average 8.67% lipids, while the cheeses with ALP addition from 8.75 to 8.80% (Figure 1). The addition of ALP did not significantly affect the lipids content of the experimental samples. The tested cheeses had a normative fat content typical for the full-fat ACCs [36]. ACCs had a stable lipids content during storage (Table 1), and the changes observed did not exceed 0.2%. Astaxanthin was recovered from shrimp shells along with the lipid fraction in which carotenoids are perfectly soluble. Lipid preparations from shrimp shells contain polyunsaturated fatty acids (including DHA and EPA), α-tocopherol, cholesterol and therefore has recently been proposed as a natural food ingredient [37]. The recommended acceptable daily intake (ADI) of astaxanthin should not exceed 0.034 mg/kg body weight, or 2.38 mg per day by a 70 kg human [38]. Astaxanthin is not the main component of the extract, as it is reported to range between 2 and 5 mg/g lipid extract [39]. Consumption of 100 g of the ACC with 1% ALP will not exceed the ADI for astaxanthin. Astaxanthin absorption occurs only in the presence of fatty acids and bile salts and is limited by the presence of dietary fibre. Together with lipids, the addition of ALP respectively increased the astaxanthin content. One hundred grams of ACC with ALP-0.25% contained 153 ± 5 µg astaxanthin, with ALP-0.5% 294 ± 13 µg, and with ALP-1% 582 ± 26 µg.

**Figure 2:**
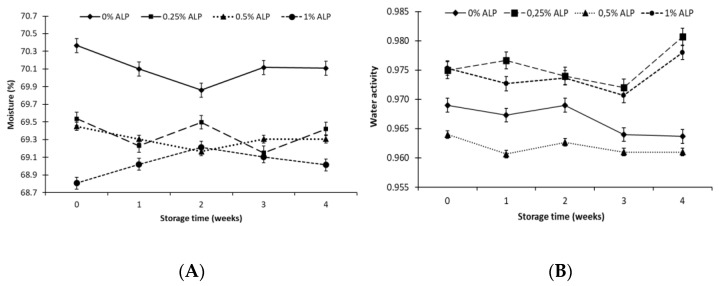
Moisture content (**A**) and water activity (**B**) in acid curd cheese with different addition (0–1%) of astaxanthin lipid preparation (ALP) during 4 weeks of refrigerated storage (5 ± 1 °C).

All the evaluated cheeses were characterised by stable and the normative moisture content ranging from 68.8% to 70.4% (Figure 2A and Table 1). The control sample had 70.4% moisture. The increase in the lipid content by the addition of ALP was accompanied by a decrease in the moisture of the cheeses to 68.8–69.5%. Changes in moisture content of ACCs were statistically insignificant. Such small changes in moisture are probably due to evaporation of water from the product to the package walls observed in refrigerated stored foods. The lack of significant changes in the chemical composition of acid curd cheeses stored under refrigeration was reported, among others, by Śmietana et al. [40].

Changes in water activity (a_w_) of the test samples during storage were not statistically significant (Table 1). However, a_w_ was significantly affected by the addition of ALP. Cheese with 0.25% ALP had the highest average water activity of 0.25% (a_w_ = 0.976), followed by ALP 1% (0.974), a control sample (0.967) and ALP 0.5% (0.962) (Figure 2B). Similar water activity values of tvarog were shown by Godula et al. [41], who did not report any significant change in a_w_ during storage stability studies of traditional and lactose-free acid curd cheese. Water activity expresses the degree of water availability to microorganisms and depends on, for example, salt content, binding of water by food components, as well as acidity [42,43]. Water activity affecting shelf-life, fat oxidation, protein denaturation, and vitamin degradation. Alla El-Bialy and Abd El-Khalek [44] found that the addition of astaxanthin preparations to tilapia meat reduced a_w_ by 0.05 and this effect was maintained for 20 days of storage; however, phenomena in fish meat can be different than this one in tvarog. Statistical analysis showed that there was no correlation between moisture and a_w_ in the tvarogs. The correlation coefficient for samples 0%, 0.25%, 0.5% and 1% was −0.001, 0.27, 0.33 and −0.40 respectively.

**Figure 3:**
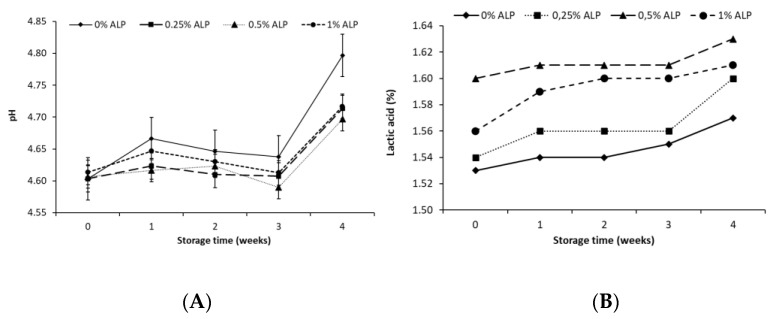
Value of pH (**A**) and titratable acidity (**B**) of acid curd cheese with different addition (0–1%) of astaxanthin lipid preparation (ALP) during 4 weeks of refrigerated storage (5 ± 1 °C).

The addition of astaxanthin significantly affects the pH value of the ACCs (Figure 3, Table 1). During 4 weeks of storage, the pH value of the ACCs varied significantly (*p* < 0.05) between 4.58 and 4.80. Cheeses with ALP had the lower pH values in the range of 4.63–4.64. The highest pH during storage was found in the control sample (4.67). Acid curd cheese, namely fresh cheese, has a pH value close to the isoelectric point of casein, i.e., 4.4–4.6 [41]. These pH values guarantee the correct curd formation and the separation of the whey [45]. The ACC pH is also related to the presence of competing microorganisms, inhibitory substances, technological treatments [41], differences in moisture and lactose content. Variation in vitamin and amino acid contents can modify the development of starter bacteria, which is related to the proteolytic activity of the bacteria [22].

The titratable acidity of a fresh tvarog (a control sample) on the day of starting the tests was 1.53% (Figure 3B). For every one percent of ALP addition, the acidity of the ACC significantly increased by 0.7% lactic acid, except for the difference between the control sample and the tvarog with the lowest ALP content. During storage, the acidity of all samples increased. For the control sample, the acidity increased by 0.4% after 4 weeks, with a characteristic stabilization between the 1st and 2nd weeks of storage. The addition of ALP prolonged the acidity stabilization time in the ACCs by one week (to the 3rd week). This may indicate the bacteriostatic properties of astaxanthin against lactic acid bacteria [44]. During 4 weeks of storage, the acidity least increased by 0.3% in the ACC containing 0.5% ALP, while it increased by 0.5–0.6% in the other samples. Correlation analysis showed that the pH value almost fully positively correlates (r = 0.917–0.944) with the acidity of the ACCs containing 0% and 0.25% ALP. The addition of 0.5% and 1% ALP reduced the value of the correlation coefficient to 0.889 and 0.580, respectively. This may confirm the inhibitory effect on lactic acid production by bacteria and/or the buffering effect of the substances contained in the astaxanthin preparation. The statistical analysis confirmed the significant effect of the storage time and ALP addition on the titratable acidity of the ACC.

**Figure 4:**
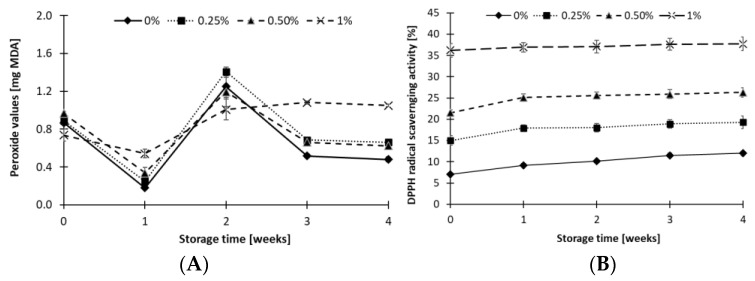
Peroxide value (**A**) and antioxidant activity measured by the DPPH scavenging assay (**B**) of acid curd cheese with different addition (0–1%) of astaxanthin lipid preparation (ALP) during 4 weeks of refrigerated storage (5 ± 1 °C). Antioxidant activity is expressed as percent of scavenging activity.

Peroxide value (PV) in the control sample was 0.87 mg MDA (Figure 4A). The addition of ALP in amounts of 0.25% and 0.5% did not significantly affect the content of lipid peroxidation products in ACCs. Only the addition of 1% ALP reduced the PV of tvarog to 0.74 mg MDA. During 3 weeks of storage, the content of secondary lipid peroxidation products in the control samples and those containing 0.25–0.5% ALP alternately decreased and increased, and then stabilized over a period of 3–4 weeks. In these ACCs, after 3 and 4 weeks of storage, the PV values were lower than before refrigerated storage of the samples. The average PV value in all samples was 0.77 mg MDA, indicating a very low level of lipid oxidation. For the ACC with the highest ALP addition, the PV value during storage changed more in accordance with a linear function and stabilised after the second week. It is likely that a 1% ALP addition increases both the amount of astaxanthin and unsaturated fatty acids, which in the protein–lipid matrix of the ACCs undergo a change in the stability equilibrium of antioxidant capacity and lipid oxidation compared to lower doses of ALP. On the other hand, the smaller variation of PV change during storage of tvarog with 1% ALP addition may indicate the effective antioxidant activity of astaxanthin. Gómez-Estaca et al. [37], after 120 days of storage of a lipid extract of astaxanthin at room temperature, observed little lipid oxidation, manifested by a slight decrease in omega-3 fatty acids, and found no accumulation of TBARS or formation of oxidised forms of cholesterol.

The antioxidant activity of the control sample was 7% (Figure 4B). The samples with ALP had higher scavenging activities than the control sample (*p* < 0.05). Radical scavenging activities (RSA) increased significantly (*p* < 0.05) in all samples with increased storage time. In the control sample, RSA increased by 5% after 4 weeks of storage and the respective increase in 0.25%, 0.50% and 1% ALP cheese was 4.3%, 4.8% and 1.5%, respectively. An increase in DPPH during the ACC storage was also shown by Kariyawasam et al. [46]. Cheese contains a large amount of casein, which is proteolyzed by enzymes present in milk and bacterial enzymes, causing the production of peptides, some of which show a potential antioxidant activity [47]. In the present study, this potential was significantly increased in the presence of astaxanthin. UV–VIS analysis of chloroform extracts showed that only 1% ALP addition significantly increased both primary and secondary lipid oxidation products in the ACC (data not shown). Moreover, with increasing ALP addition, there were more primary oxidation products than secondary oxidation products.

**Figure 5:**
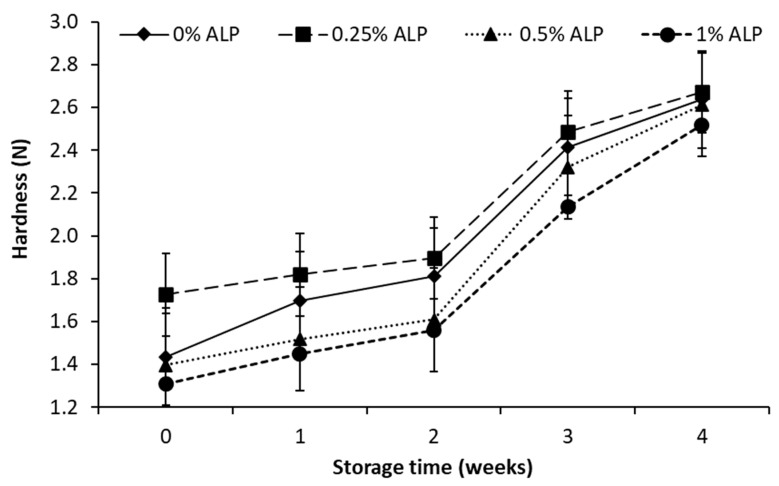
Hardness of acid curd cheese with different addition (0–1%) of astaxanthin lipid preparation (ALP) during 4 weeks of refrigerated storage (5 ± 1 °C).

The hardness of the ACC depends on the composition, proteolytic activity and interaction between the components. Changes in acidity determine the degree of decalcification of the curd and thus the disintegration of the casein micelles. This process has a decisive influence on the basic structure of the ACC and the ratio of water to casein content. The addition of ALP significantly affected the hardness of the ACC tested, which was 1.3 –1.7 *N* (Figure 5, Table 1). Hardness correlated very strongly positively with acidity (r = 0.65–0.96), which may indicate that an increase in the amount of lactic acid in the curd increases hardness by intensifying the curd shrinkage and increasing whey syneresis. However, the hardness of ACC with 1% ALP was lower by 0.12–0.28 *N* than ACC without ALP addition. Presumably, lipids from ALP (especially branched unsaturated fatty acids) prevented protein binding and syneresis. With a longer storage period, the differences in the hardness between the samples decreased. After 2 weeks of storage, the ACC hardness increased to 1.6–1.9 *N*. During another 2 weeks, the hardness increased faster and at the end of the storage period it was 2.5–2.7 *N*. Statistical analysis confirms a significant effect of storage time and showed that hardness correlated very weakly positively with moisture content and weakly negatively with lipid oxidation level.

The colour parameters of the control ACC, especially L* = 94.8, were typical (Table 2) [48,49]. The ALP addition decreased the brightness by 2.6, 3.9 and 6.4 units, respectively, according to a logarithmic curve. During storage of the control sample, *L* * decreased by 4 units after 3 weeks and by 3, 2 and 0.4 units in cheese with ALP. The redness parameter (a*) of the control sample was 1.6. The addition of ALP increased the redness of colour by 0.9, 2.4 and 5.1 respectively, and the differences between samples increased according to a power function. Storage increased of parameter *a* * by 0.5–0.7 units. In the case of the yellowness parameter (b*), its value in the control sample was 9.7 and higher by 2, 3.3 and 5.7 units in cheeses with ALP, respectively.

The differences in yellowness between the samples increased according to a logarithmic function. Refrigerated storage increased the b* value in all the ACC by 0.45–0.55. The calculated colour difference (ΔE) between the control sample and the samples with added ALP was 2.57, 5.08 and 9.55, respectively.

These results show that consumers will notice the difference in tvarog colour with the naked eye, even when 0.25% ALP is added. Also, the colour difference between 0.25% ALP and 0.5% ALP samples was high (2.68). The results show that higher ALP concentration increased the colour difference (ΔE) between samples according to the logarithmic function. The colour parameters of the ACCs with ALP were more stable during storage than parameters in the control cheese.

Additional analysis of colour saturation (C) showed that the ACCs with ALP had higher colour saturation and storage time increased the difference in saturation compared to the control sample (Table 2). Higher colour saturation value is perceived as better colour purity. On the other hand, the colour hue (h) increased with increasing ALP additive concentration according to a power function like the a* parameter. During storage, the colour hue of the ACC decreased by the same value in all samples (Table 2). These results show that the positive effect of ALP additive on colour was progressively greater with longer storage time of cheeses, which was not the case with the control tvarog.

**Figure 6:**
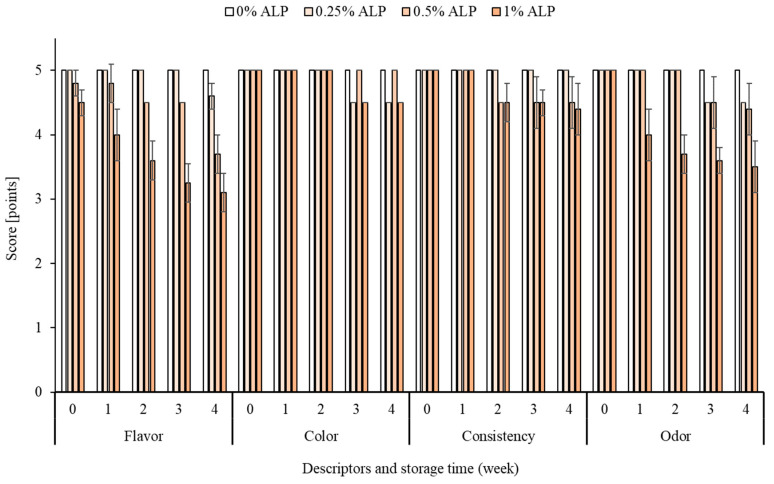
Sensory evaluation 4 descriptors of acid curd cheese with different addition (0–1%) of astaxanthin lipid preparation (ALP) during 4 weeks of refrigerated storage (5 ± 1 °C).

The highest sensory score of the ACC was given to the control sample, which had maximum scores for all descriptors during 4 weeks of storage (Figure 6). Maximum scores were given to all samples for colour, which was homogeneous and favourably acceptable despite the different addition of ALP. However, the panellists pointed out that the salmon colour for ALP 0.25% was too light. The ACCs with 0.25% and 0.5% ALP addition received the most similar scores for flavour and odour up to the 3rd week of storage. After the 4th week of storage, little difference in flavour and odour was found between the samples. The cheese with 1% ALP had the lowest scores for flavour and odour. Already on day 0, the cheese had an odour originating from the astaxanthin preparation, absent in the other samples. During cold storage, sensory analysis showed a gradual deterioration of the flavour and odour of the sample with 1% ALP added. After the 3rd and 4th weeks of storage, an unusual fishy aftertaste and grassy odour was perceptible in this sample. All samples with ALP had a stable structure and consistency, although a slight increase in firmness was observed after 2 weeks of storage. Considering the favourable salmon colour from ALP and, on the other hand, the sensory characteristics, the optimal sample was the ACC with 0.5% ALP. Overall sensory quality were: the control sample 5.0, ALP 0.25–4.9%, ALP 0.5–4.7% and ALP 1–4.3% (Figure 6).

## 4. Conclusions

The results showed that new functional characteristics of acid curd cheese can be obtained using astaxanthin lipid preparation (ALP), which was obtained from shrimp shell by-products. The acid curd cheeses with ALP have higher antioxidant activity and lower amounts of lipid oxidation products for the highest ALP addition. The addition of ALP changed the colour of the acid curd cheese from white to salmon colour, which was highly preferred by panel members. Objective colour parameters were more stable, and values changes more favourable during cold storage, compared to the cheese without astaxanthin preparation. The addition of ALP resulted in changes in the acidity, pH, water activity and hardness of the cheeses. It is likely that astaxanthin in free/dissolved (non-encapsulated) form could interact with other cheese components. The results of sensory analysis confirmed that the most favourable addition of ALP to the acid curd cheese was 0.5%.

## Figures and Tables

**Table 1:** Results of 2-way ANOVA of physicochemical properties and overall sensory quality of acid curd cheese (ACC). *p* value with * means that differences are statistically significant (F > Test F).

Parameter	Factor	F	*p*	Test F
Lipids content	storage time	0.229	0.921	2.606
	cheese variant	1.867	0.151	2.839
	interaction	0.248	0.994	2.003
Moisture	storage time	0.471	0.757	2.606
	cheese variant	9.106	˂0.001 *	2.839
	interaction	1.396	0.208	2.003
pH	storage time	24.633	˂0.001 *	2.606
	cheese variant	4.626	0.007 *	2.839
	interaction	0.772	0.675	2.003
Titratable acidity	storage time	3.181	0.023 *	2.606
	cheese variant	17.166	˂0.001 *	2.839
	interaction	0.297	0.986	2.003
Water activity	storage time	1.840	0.140	2.606
	cheese variant	42.347	˂0.001 *	2.839
	interaction	1.436	0.190	2.003
Peroxide values	storage time	70.490	˂0.001 *	2.606
	cheese variant	8.313	˂0.001 *	2.839
	interaction	30.914	˂0.001 *	2.003
Antioxidant activity	storage time	14.581	˂0.001 *	2.606
	cheese variant	143.215	˂0.001 *	2.839
	interaction	7.032	˂0.001 *	2.003
Hardness	storage time	169.915	˂0.001 *	2.486
	cheese variant	16.977	˂0.001 *	2.718
	interaction	0.687	0.759	1.875
L*	storage time	18.811	˂0.001 *	2.486
	cheese variant	14.251	˂0.001*	2.719
	interaction	3.1874	˂0.001 *	1.875
a*	storage time	7.658	˂0.001 *	2.486
	cheese variant	406.139	˂0.001 *	2.719
	interaction	1.510	0.138	1.875
b*	storage time	4.258	˂0.001 *	2.486
	cheese variant	79.259	˂0.001 *	2.719
	interaction	3.262	˂0.001 *	1.875
h	storage time	3.142	˂0.001 *	2.486
	cheese variant	1.532	˂0.001	2.719
	interaction	0.135	0.208	1.875
C	storage time	14.751	˂0.001 *	2.486
	cheese variant	2.934	˂0.001 *	2.719
	interaction	1.421	˂0.001	1.875
Overall sensory quality	storage time	8.476	˂0.001 *	2.557
	cheese variant	1.627	˂0.001	2.557
	interaction	0.823	˂0.001	1.850

**Table 2:** Results of means and standard deviations for L* a* b* parameters, saturation (C) and hue (h) of colour.

Sample	Week	L*	a*	b*	h	C
0% ALP	0	94.84 ± 0.09	1.64 ± 0.49	9.71 ± 0.11	1.40 ± 0.01	9.84 ± 0.36
	1	93.48 ± 0.71	1.70 ± 0.17	10.11 ± 0.07	1.40 ± 0.01	10.25 ± 0.21
	2	92.87 ± 0.03	1.99 ± 0.03	10.55 ± 0.46	1.38 ± 0.02	10.74 ± 0.23
	3	90.85 ± 0.63	1.86 ± 0.09	9.76 ± 0.21	1.38 ± 0.02	9.94 ± 0.18
	4	93.14 ± 0.40	2.30 ± 0.26	10.16 ± 0.27	1.38 ± 0.01	10.42 ± 0.15
0.25% ALP	0	92.21 ± 0.67	2.58 ± 0.02	11.72 ± 0.40	1.35 ± 0.01	12.00 ± 0.32
	1	91.85 ± 0.17	2.56 ± 0.10	11.47 ± 0.16	1.35 ± 0.03	11.75 ± 0.24
	2	91.36 ± 0.45	2.65 ± 0.08	11.40 ± 0.18	1.34 ± 0.01	11.74 ± 0.41
	3	89.26 ± 0.62	2.46 ± 0.03	11.85 ± 0.57	1.37 ± 0.01	12.10 ± 0.21
	4	91.68 ± 0.85	2.53 ± 0.09	12.22 ± 0.45	1.37 ± 0.02	12.52 ± 0.31
0.50% ALP	0	90.94 ± 0.75	3.99 ± 0.16	13.02 ± 0.30	1.27 ± 0.02	13.62 ± 0.25
	1	90.46 ± 0.21	4.09 ± 0.21	13.02 ± 0.28	1.27 ± 0.01	13.65 ± 0.13
	2	88.80 ± 0.23	4.55 ± 0.16	13.21 ± 0.19	1.24 ± 0.03	13.97 ± 0.41
	3	88.99 ± 0.49	3.99 ± 0.17	13.23 ± 0.40	1.28 ± 0.01	13.82 ± 0.20
	4	90.13 ± 0.38	4.52 ± 0.22	13.49 ± 0.17	1.25 ± 0.03	14.23 ± 0.25
1.0% ALP	0	88.48 ± 0.59	6.69 ± 0.35	15.35 ± 0.81	1.16 ± 0.01	16.77 ± 0.12
	1	87.48 ± 0.23	7.25 ± 0.23	15.85 ± 0.24	1.14 ± 0.02	17.43 ± 0.31
	2	86.70 ± 0.88	7.40 ± 0.53	15.38 ± 0.45	1.12 ± 0.05	17.07 ± 0.23
	3	88.11 ± 0.51	7.22 ± 0.60	16.64 ± 0.95	1.16 ± 0.01	18.14 ± 0.15
	4	88.06 ± 0.85	7.40 ± 0.73	15.89 ± 0.72	1.14 ± 0.01	17.53 ± 0.13

## Data Availability

The data presented in this study are available on request from the corresponding author.
